# Sulfur Mustard and Immunology; Trends of 20 Years Research in the Web of Science Core Collection: A Scientometric Review

**DOI:** 10.18502/ijph.v49i7.3573

**Published:** 2020-07

**Authors:** Ramin SALOUTI, Roghayeh GHAZAVI, Sattar RAJABI, Mohammad ZARE, Mohammadreza TALEBNEJAD, Mohammad Bagher ABTAHI, Maryam PARVIZI, Sedigheh MADANI, Fahimeh ASADI-AMOLI, Ensieh-Sadat MIRSHARIF, Reza GHAREBAGHI, Fatemeh HEIDARY

**Affiliations:** 1. Poostchi Ophthalmology Research Center, Department of Ophthalmology, School of Medicine, Shiraz University of Medical Sciences, Shiraz, Iran; 2. Salouti Eye Research Center, Salouti Eye Clinic, Shiraz, Iran; 3. Vice Chancellery of Research and Technology, Isfahan University of Medical Sciences, Isfahan, Iran; 4. Department of Optometry, School of Rehabilitation, Iran University of Medical Sciences, Tehran, Iran; 5. Labbafinejad Hospital, Shahid Beheshti University of Medical Sciences, Tehran, Iran; 6. Department of Pathology, Mofid Hospital, Shahid Beheshti University of Medical Sciences, Tehran, Iran; 7. Endocrinology and Metabolism Research Institute, Tehran University of Medical Sciences, Tehran, Iran; 8. Department of Pathology, Farabi Eye Hospital, Tehran University of Medical Sciences, Tehran, Iran; 9. Immunoregulation Research Center, Shahed University, Tehran, Iran; 10. Kish International Campus, University of Tehran, Tehran, Iran; 11. Ophthalmology Division, Taleghani Hospital, Ahvaz Jundishapur University of Medical Sciences, Ahvaz, Iran

**Keywords:** Sulfur mustard, Immunology, Scientometric review

## Abstract

**Background:**

We aimed to use the scientometric approach to evaluate immunological studies on the subject of sulfur mustard over the past 20 years.

**Methods:**

In this scientometric study, the Web of Science Core Collection was searched on the studies about sulfur mustard. The published papers related to the field of immunology were retrieved from these papers. HistCite software and VOSviewer were the applied software packages for bibliometric analysis, information visualization, and creating bibliometric networks.

**Results:**

Over the past 20 years, 741 researchers from 22 countries have published 201 scientific papers in 95 journals. Iran and the United States with 93 and 68 published articles ranked at the top. The Journal of International Immunopharmacology, with 33 published papers, 439 Total Global Citation Score (TGCS), and 105 Total Local Citation Score (TLCS) was the most productive and most influential in this regard. The paper entitled “Biomonitoring of exposure to chemical warfare agents: A review” and another paper entitled “Sardasht-Iran Cohort Study of Chemical Warfare Victims: Design and Methods” were the most influential papers in this topic with 200 TGCS and 27 TLCS, respectively. The most productive and the most influential centers were “Immunoregulation Research Center of Shahed University” and “The Janbazan Medical and Engineering Research Center (JMERC),” respectively.

**Conclusion:**

The result of our report as the unique scientometric evaluation of the research on sulfur mustard and Immunology can be used as a roadmap for authors, researchers, and policymakers to define the best ways to allocate their financial and executive resources.

## Introduction

Sulfur Mustard, bis (2-chloroethyl) sulfide, has been used in the conflicts as a chemical warfare agent since the early 20^th^ century. It was also used during the Iran-Iraq war against Iranian soldiers and civilians that resulted in more than 100,000 chemical casualties. It is an extremely reactive alkylating agent (1–4). In general, the most commonly compromised organs following exposure to this chemical warfare are lungs, eyes, and skin, although the involvement of other organs has been reported (1,4). Several research studies have been conducted on its acute and chronic side effects and their management. These studies comprised in vitro studies, animal studies, and human studies from different countries all over the world, including its immunological consequences and manifestations (4).

Scientometrics is defined as “the study of the measurement of scientific and technological progress” and has a strong application-oriented tradition (5–7). Moreover, the assessment of the performance of scientific groups/researchers has increasingly been associated with their scientific achievements, so there has recently been a growing interest in scientometric evaluations (8,9).

According to our literature review, there is no scientometric study regarding global trend and status of immunological studies conducted in the subject of sulfur mustard. Therefore, we used a scientometric approach to evaluate immunological studies on the subject of sulfur mustard over the past 20 years.

## Methods

We searched Web of Science Core Collection to find out the published papers with the purpose of conclusive search on the sulfur mustard (“Mustard gas” OR “Di-2-chloroethyl Sulfide” OR “Di 2 chloroethyl Sulfide” OR “Dichlorodiethyl Sulfide” OR “Sulfur Mustard” OR “Yellow Cross Liquid” OR Yperite OR “Bis (beta-chloroethyl) Sulfide” OR Mustardgas OR Psoriazin OR “2-chloroethyl ethyl sulfide”). Next, the articles related to the field of immunology were retrieved from these papers using these two following methods. In the first method, the papers on the sulfur mustard subject were restricted to the Immunology category by using the Web of Science categories limit. In the second method, the immun* term was added to the search strategy regarding the sulfur mustard. The “*” or Truncation sign is included at the end of this term to retrieve all words related to this topic. In the end, the retrieved papers are combined using both methods. To warrant that there is a maximum relationship between our search subject and literature, keywords were searched only in the title of the papers. We analyzed the published papers in June 2019, including original papers, review articles, letters, and editorials over the past 20 years (1999–2018).

Scientific papers identified in this field were analyzed using different scientometric indices that included the most active authors, journals, countries, institutions, and research centers (referring to the number of published papers) as well as the most influential authors, journals, countries, institutions and research centers (referring to the citations of published articles). The trend of the number of published papers and their citations were evaluated in distinct years, besides calculation of the growth factor. Furthermore, the clustering of scientific topics was assessed. Applied software package for bibliometric analysis and information visualization, as well as creating and visualizing bibliometric networks, were HistCite and VOSviewer mapping software of Centre for Science and Technology Studies, Leiden University, The Netherlands. The impact factors of the journals were retrieved via the Journal Citation Reports (JCR) database, provided by ISI-Thomson Reuters database (Thomson Reuters, New York, NY).

## Results

### Total Number of Published Papers

**Fig. 1:**
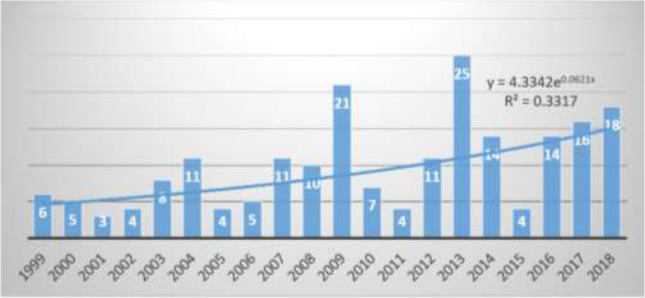
The number of published papers per year in the past 20 years in the field of sulfur mustard and Immunology (1999–2018)

We found quite a good number of published papers on sulfur mustard and Immunology. Overall, 201 articles were met search criteria and included in the final analysis. Figure 1 shows a changing trend in the number of published papers over the past 20 years with peaks in 2009 and 2013 though the exponential increase is noted since 2015 onwards. The highest and lowest number of published papers during this 20 years was in 2013, with 25 papers and 2001, with 3 papers, respectively. The overall growth rate of products was 6.21%.

### Total Number of Citations

**Fig. 2:**
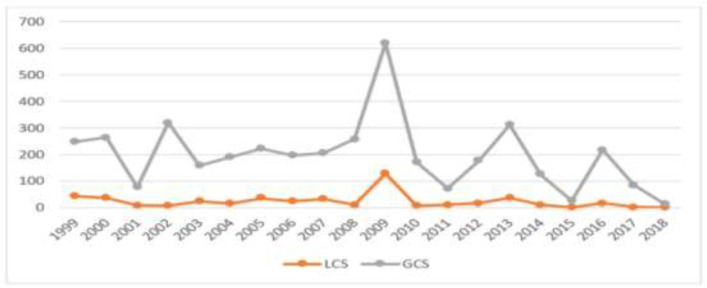
Frequency of Total of Global Citation Scores (TGCS) and the Total of Local Citation Scores (TLCS) of the published papers per year in the past 20 years in the field of sulfur mustard and Immunology (1999–2018)

Overall, 201 published papers related to sulfur mustard and Immunology with the sum of times cited equals to 3485 were found on ISI-Web of Science Database. Whereby, times cited without self-citation was 2927 and in general 1709 papers cited sulfur mustard and Immunology papers in which the citing papers without self-citation was 1552. The mean number of citation per item was 17.34, and the overall H-index was 34. These findings were derived from an index called Total of Global Citation Scores (TGCS) in ISI-Web of Science Database and the Total of Local Citation Scores (TLCS) (10). Figure 2 shows the trend of TGCS and TLCS over the past 20 years in this area of research.

A changing trend in the number TGCS is evident, which almost always was higher than that in TLCS. However, the frequency of TLCS citations, except the 2009 peak, in different years was constant and less than 100 citations. Five papers with more than 70 TGCS were “Biomonitoring of exposure to chemical warfare agents: A review,” “Nanoparticle-Based Electrochemical Immunosensor for the Detection of Phosphorylated Acetylcholinesterase: An Exposure Biomarker of Organophosphate Pesticides and Nerve Agents,” “The chronic effects of sulfur mustard exposure,” “Chronic and delayed-onset mustard gas keratitis - Report of 48 patients and review of literature,” and “Immunoglobulins and cellular constituents of the BAL fluid of patients with sulfur mustard gas-induced pulmonary fibrosis.”

Table 1 presents the published papers with more than 50 TGCS, which are 12 published papers. Three papers with more than 15 TLCS were “Sardasht-Iran Cohort Study of Chemical Warfare Victims: Design and Methods,” “Immunoglobulins and cellular constituents of the BAL fluid of patients with sulfur mustard gas-induced pulmonary fibrosis,” and “Alterations in serum levels of inflammatory cytokines (TNF, IL-1alpha, IL-1beta, and IL-1Ra) 20 years after sulfur mustard exposure: Sardasht-Iran cohort study.” The published papers with ≥10 TLCS, which are 13 published papers. The paper entitled “Biomonitoring of exposure to chemical warfare agents: A review” was published in 2002 in the Journal of Toxicology and Applied Pharmacology with 200 TGCS and paper entitled “Sardasht-Iran Cohort Study of Chemical Warfare Victims: Design and Methods” published in 2009 in the journal of Archives of Iranian Medicine with 27 TLCS were the most influential ones.

**Table 1:** The published papers in the subject area of sulfur mustard and Immunology with the highest amount of Total of Global Citation Scores (more than 50 TGCS) in ISI-Web of Science Database

***No***	***Author***	***Title***	***Year***	***TGCS***
1	Noort, D et al.	Biomonitoring of exposure to chemical warfare agents: A review	2002	200
2	Liu, GD et al.	Nanoparticle-Based Electrochemical Immunosensor for the Detection of Phosphorylated Acetylcholinesterase: An Exposure Biomarker of Organophosphate Pesticides and Nerve Agents	2008	93
3	Rowell, M et al.	The chronic effects of sulfur mustard exposure	2009	80
4	Javadi, MA et al.	Chronic and delayed-onset mustard gas keratitis - Report of 48 patients and review of literature	2005	75
5	Emad, A et al.	Immunoglobulins and cellular constituents of the BAL fluid of patients with sulfur mustard gas-induced pulmonary fibrosis	1999	71
6	Sabourin, CLK et al.	Alterations in inflammatory cytokine gene expression in sulfur mustard-exposed mouse skin	2000	69
7	Ricketts, KM et al.	Inflammatory cytokine response in sulfur mustard-exposed mouse skin	2000	68
8	Kumar, V et al.	A Selective Turn-On Fluorescent Sensor for Sulfur Mustard Simulants	2013	67
9	Ghazanfari, T et al.	Sardasht-Iran Cohort Study of Chemical Warfare Victims: Design and Methods	2009	58
10	Hefazi, M et al.	Delayed complications of sulfur mustard poisoning in the skin and the immune system of Iranian veterans 16–20 years after exposure	2006	56
11	Mahmoudi, M et al.	Long-term hematological and immunological complications of sulfur mustard poisoning in Iranian veterans	2005	55
12	Arroyo, CM et al.	Response of normal human keratinocytes to sulfur mustard: Cytokine release	2000	52

### Papers Distribution by Country and Language

Twenty-two countries were the countries that published the papers in sulfur mustard and Immunology research field; Iran with 93 papers, the United States with 68 articles, and Germany with 11 articles were the three top countries in this area. The most influential countries based on TGCS and TLCS were the United States with 1571 TGCS and 129 TLCS and Iran with 1429 TGCS and 295 TLCS. The majority of published papers in this area were in English.

### The Top Journals, Institutions, Research Centers, and Authors

Overall, 95 journals had published papers related to this field for over 20 years. Of them, the Journal of International Immunopharmacology with 33 published papers, was the most productive journal in this field. Journals with six or more published papers were defined as the journals with the highest productive journals, of which 7 journals had this criterion. Table 2 presented that the Journal of International Immunopharmacology with 439 TGCS and 105 TLCS was the most influential journal.

**Table 2:** Journals with the highest number of published papers (with ≥ 6 papers), the highest number of Total of Global Cita-tion Scores (with more than 100 TGCS) and Total of Local Citation Scores (with more than 20 TLCS) in the Subject Area of the sulfur mustard

***Number***	***Journal***	***Number of Published Papers***	***Journal***	***TGCS***	***Journal***	***TLCS***
1	International Immunopharmacology	33	International Immunopharmacology	439	International Immunopharmacology	105
2	Iranian Journal of Allergy Asthma And Immunology	10	Toxicology and Applied Pharmacology	300	Journal of Applied Toxicology	39
3	Journal of Applied Toxicology	10	Journal of Applied Toxicology	247	Archives of Iranian Medicine	27
4	Toxicology and Applied Pharmacology	8	Toxicology	190	Iranian Journal of Allergy Asthma and Immunology	25
5	Toxicology Letters	7	International Journal of Cancer	126	Toxin Reviews	22
6	Immunopharmacology and Immunotoxicology	6			Chest	21
7	Toxicology	6				

Totally, 217 research institutes and 399 research centers are active in this research field. Twenty-one of these centers have published more than 5 papers in this area. The most productive center was the Immunoregulation Research Center of Shahed University with 27 papers followed by Baqiyatallah Univercity of Medical Sciences Chemical Injuries Research Center (26 papers) and The Janbazan Medical and Engineering Research Center (24 papers). The most influential center was The Janbazan Medical and Engineering Research Center (JMERC) with 326 TGCS and 125 TLCS.

Overall, 741 authors from 22 countries contributed to the field, 7 of whom have published over 15 papers (Table 3). The most productive author was Ghazanfari T. with 30 papers from Immuno-regulation Research Center of Shahed University, Tehran, Iran. Out of 741 authors, 9 authors had TGCS more than 250, and 5 authors had TLCS more than 100 listed in Table 3. Amongst them, Hassan ZM. from the Department of Immunology, Faculty of Medical Sciences, Tarbiat Mo-dares University, Tehran, Iran, with 390 TGCS and 129 TLCS was the most influential researcher in this field.

**Table 3:** Authors with the highest number of published papers (with more than 15 papers), the highest number of Total of Global Citation Scores (with more than 250 TGCS), and the highest number of Total of Local Citation Scores (with more than 100 TLCS) in the subject area of the sulfur mustard and Immunology

***Number***	***Author***	***Number of Published Papers***	***Author***	***TGCS***	***Author***	***TLCS***
1	Ghazanfari T	30	Hassan ZM	390	Hassan ZM	129
2	Hassan ZM	29	Ghanei M	368	Ghazanfari T	127
3	Ghanei M	26	Ghazanfari T	341	Soroush MR	125
4	Faghihzadeh S	24	Soroush MR	326	Faghihzadeh S	124
5	Soroush MR	24	Faghihzadeh S	319	Yaraee R	118
6	Yaraee R	20	Yaraee R	294		
7	Panahi Y	15	Noort D	293		
			Benschop HP	274		
			Casillas RP	266		

### Subjects Trend

**Fig. 3:**
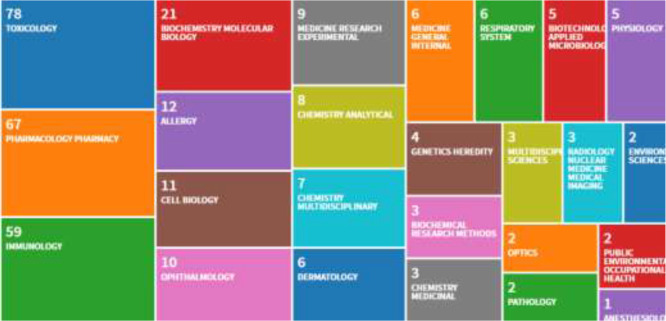
25 ISI-Web of Science categories with the most number of published papers in the field of sulfur mustard and Immunology

**Fig. 4:**
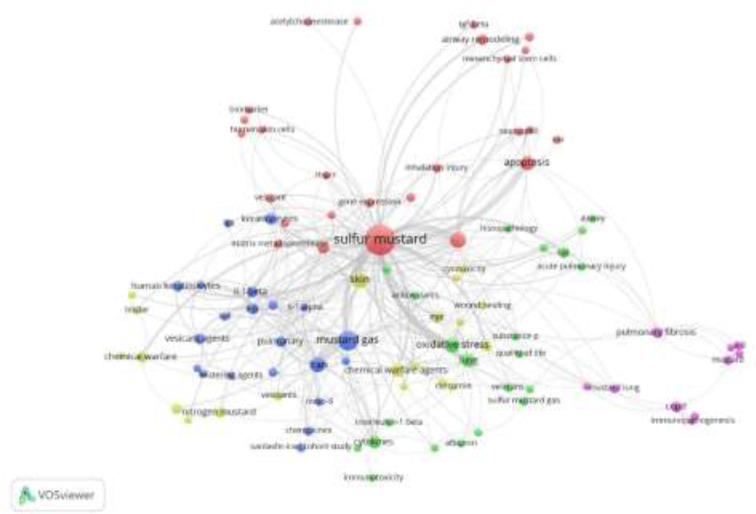
Subject distribution and their clustering in the field of the “Sulfur Mustard and Immunology” for published papers between 1999 and 2018. Dot size is proportional to the number of published articles on that subject. The lines between the subjects indicate a relationship between those subjects and the thickness of the line indicate the strength of the link

The subject trend distribution was assessed using “co-occurring subject category indicator” and “page rank” on the ISI-Web of Science Core Collection. Figure 3 illustrated 25 ISI-Web of Science categories with the most number of published papers in the field of sulfur mustard and Immunology. Figure 4 illustrates the subject distribution and their clustering in the field of the sulfur mustard and Immunology for the published papers between 1999 and 2018. Dot size is proportional to the number of published papers on that subject. The lines between the subjects indicate a relationship between those subjects and the thickness of the line indicate the strength of the link.

## Discussion

The scientometric evaluation in the present study showed that over the past 20 years, 741 researchers from 22 countries had published 201 scientific papers in 95 journals. Iran and the United States with 93 and 68 published articles ranked among the top countries on the issue of sulfur mustard and Immunology. The Journal of *International Immunopharmacology*, with 33 published papers, 439 TGCS, and 105 TLCS was the most productive and most influential journal among 95 journals. Ghzanfari T., published 30 articles, was the most productive and Hassan ZM. with 390 TGCS and 129 TLCS was the most influential researcher among active researchers in this field.

The paper entitled “Biomonitoring of exposure to chemical warfare agents: A review” published in 2002 in the *Journal of Toxicology and Applied Pharmacology* with 200 TGCS and paper entitled “Sardasht-Iran Cohort Study of Chemical Warfare Victims: Design and Methods” published in 2009 in the journal of *Archives of Iranian Medicine* with 27 TLCS were the most influential among 201 published papers. The most productive and the most influential center were the Immunoregulation Research Center of Shahed University with 27 papers and the Janbazan Medical and Engineering Research Center (JMERC) with 326 TGCS and 125 TLCS, respectively. In a recent scientometric evaluation three databases of ISI, Medline, and Scopus were reviewed between 1988 and 2012, to examine the published papers on the mustard gas in the Iran-Iraq conflict (11). Ninety articles have been published on this topic. In the current study, a scientometric evaluation on studies regarding mustard gas was conducted, and one database, ISI, was searched. Compared to the previous study, our search was more conclusive as it included all published papers regarding mustard gas–not just Iran-Iraq conflict and Ghanei M. was the most productive researcher who had more than 20 published papers. In our study, the process of publishing papers was examined throughout the past 20 years, with 201 papers, including original papers, review papers, letters to the editors, and editorial papers, of which 93 were from Iran. Moreover, in the current study, Ghzanfari T. was the most productive, and Hassan ZM. was the most influential researcher among active researchers in this field. The results of our study indicate that the citations to the published papers of Hassan ZM were 390 TGCS and 129 TLCS, which was not mentioned in the previous study. In their study, Balai-Mood’s paper (12) entitled “Comparison of early and late toxic effects of sulfur mustard in Iranian veterans” had the highest citations counted as 729. Whereas, in our evaluation, the paper entitled “Biomonitoring of exposure to chemical warfare agents: A review” (13) published in 2002 in *Journal of Toxicology and Applied Pharmacology* with 200 TGCS and paper entitled “Sardasht-Iran Cohort Study of Chemical Warfare Victims: Design and Methods” (2) published in 2009 in the journal of *Archives of Iranian Medicine* with 27 TLCS were the most influential ones.

There are few available studies regarding systematic review of organ complications of sulfur mustard. Razavi et al. studied on mustard gas-induced ocular injuries. Their evaluation on 114 articles which were more relevant to the main purpose, showed that mustard gas-related ocular complications are progressive in nature and some kinds of surgical interventions may be eventually required. A long-term and thorough follow-up for those affected patients is required (14).

In a review article Rowell et al. studied on the chronic effects of sulfur mustard exposure. Apart from ocular, skin and respiratory effects, the chronic effects are including the development of cancer, immunological and neuropsychiatric changes, and reproductive consequences (15). Panahi et al. studied on complications and carcinogenic effects of mustard gas in a systematic review and meta-analysis and concluded that prevalence of chronic consequences of mustard gas related skin, pulmonary and ocular effects is more than 90% and risk of carcinogenesis is noticeable. Among those three main reviews studied no evaluation on the scientometrics values had been reported (16).

In our study, as in the previous research, there has been significant growth in the field of published papers, which promises a brilliant horizon for research in this area because research eventually improves in the management of the complications caused by this hazardous chemical warfare agent.

One of the limitations of this research was its coverage in the Web of Science database. It is recommended to include the research topic indexed in other repositories such as Scopus. Despite this limitation, we do believe that the strengths of our finding, including its unique nature as well as global and updated coverage of all fields of “sulfur mustard and Immunology,” make its results remarkable.

## Conclusion

The result of our report as the unique scientometric evaluation of the research on “sulfur mustard and Immunology” can be used as a roadmap for authors, researchers, and policy-makers to define the best ways to allocate their financial and executive resources. In general, scientometric studies may evaluate the progress of science in different countries and may have an essential role in rating the universities and research centers according to their scientific outputs in this topic.

## Ethical considerations

Ethical issues (Including plagiarism, informed consent, misconduct, data fabrication and/or falsification, double publication and/or submission, redundancy, etc.) have been completely observed by the authors.
